# The Yellow Fever in New York

**Published:** 1856-09

**Authors:** 


*The Yellow Fever in New York.*—The whole number of cases of yellow fever that have occurred at quarantine this season, is 80; of which 46 have come directly from shipboard, 10 from the neighboring village, and 17 from the city; but all are traceable directly to the infection taken from ships in the harbor. In one case, three men sickened from pumping out an infected vessel from St. Thomas. The others were custom house officers, and captains of vessels, or their families, who had come up to the city, but all were returned to the Marine Hospital, where the treatment was very successful. Of the whole number, but eight persons are reported to have died. From this it will be seen that there is absolutely no fever in the city; all of it is imported, and for the last week only one case occurred within the city limits.—*Boston Med. and Surg. Journal.*

